# Supplementary material for: Distribution of Cleaved SNAP-25 in the Rat Brain, following Unilateral Injection of Botulinum Neurotoxin-A into the Striatum
Source: Int J Mol Sci. 2023 Jan 14;24(2):1685. doi: 10.3390/ijms24021685 (PMC9865012; doi:10.3390/ijms24021685)
Supplement: Supplementary file 1 [file ijms-24-01685-s001.zip › ijms-2013255-supplementary.pdf]

group	CPu ips			CPu con			GP ips			GP con			EP ipsi			EP con		
	mean	n	SEM	mean	n	SEM	mean	n	SEM	mean	n	SEM	mean	n	SEM	mean	n	SEM
2w	17.713	5	1.879	6.750	5	0.547	20.814	5	2.016	7.734	5	0.591	18.725	5	0.743	6.512	5	0.801
1m	17.708	4	1.603	7.061	4	0.279	19.325	4	2.081	8.212	4	0.984	20.130	4	4.366	6.730	4	0.457
3m	19.672	5	1.283	6.897	5	1.076	22.187	5	2.065	8.357	5	1.148	21.359	5	6.273	6.554	5	1.293
6m	17.964	5	2.005	8.327	5	1.433	19.395	5	2.273	7.092	5	1.502	19.657	5	7.078	7.039	5	1.125
9m	13.875	5	2.23	6.893	5	1.091	15.297	5	2.314	7.867	5	1.486	13.649	5	5.109	6.020	5	1.089
12m	9.589	5	1.916	3.857	5	0.281	10.286	5	0.56	3.770	5	0.411	8.195	5	1.814	3.193	5	0.377

group	SN ips			SN con			MThal ips			MThal con			VThal ips			VThal con		
	mean	n	SEM	mean	n	SEM	mean	n	SEM	mean	n	SEM	mean	n	SEM	mean	n	SEM
2w	26.734	5	2.748	15.226	5	2.262	16.732	5	1.586	8.965	5	1.001	12.740	5	1.064	7.057	5	0.911
1m	23.953	4	2.576	12.880	4	2.018	17.387	3	1.526	9.512	3	1.036	14.319	3	1.667	8.090	3	1.05
3m	29.532	5	3.4	12.723	5	2.34	17.072	5	2.715	9.580	5	1.944	15.531	5	1.981	7.750	5	1.432
6m	25.204	5	3.82	14.904	5	2.643	18.693	5	2.521	12.166	5	2.226	15.027	5	2.488	9.129	5	1.594
9m	22.641	5	2.687	10.771	5	1.729	11.743	5	2.228	9.621	5	1.911	12.350	5	2.012	8.386	5	1.1
12m	12.235	5	0.434	5.174	5	0.611	7.156	5	0.858	4.519	5	0.633	6.449	5	1.035	3.764	5	0.528

group	MC ips			MC con			Acb ips			Acb con			Pn ips			Pn con		
	mean	n	SEM	mean	n	SEM	mean	n	SEM	mean	n	SEM	mean	n	SEM	mean	n	SEM
2w	15.391	5	1.154	11.646	5	1.332	18.234	5	2.403	5.767	5	0.768	16.666	5	1.595	15.841	5	1.251
1m	14.310	4	1.757	9.175	4	0.388	16.651	5	1.749	6.432	5	0.409	15.819	4	0.873	15.055	4	0.824
3m	12.223	5	0.832	10.011	5	1.63	19.791	4	2.815	7.041	4	1.134	14.573	5	1.833	13.842	5	1.331
6m	16.504	5	3.516	12.446	5	2.904	17.469	5	2.86	9.007	5	1.749	16.997	5	2.965	16.568	5	2.937
9m	13.338	5	2.052	10.615	5	1.683	12.145	5	1.921	6.628	5	1.172	13.988	5	1.46	13.337	5	1.219
12m	7.674	5	0.559	5.992	5	0.159	10.244	5	1.843	3.852	5	0.51	10.425	5	1.608	10.134	5	1.44

group	VTA ips			VTA con			Hb ips			Hb con			Pir ips			Pir con		
	mean	n	SEM	mean	n	SEM	mean	n	SEM	mean	n	SEM	mean	n	SEM	mean	n	SEM
2w	15.534	5	1.936	10.107	5	1.753	17.441	5	1.556	16.774	5	1.45	23.368	5	3.115	7.645	5	0.692
1m	11.112	4	1.619	7.518	4	1.124	17.012	3	1.539	16.520	3	2.262	19.556	4	1.398	9.526	4	0.573
3m	13.558	5	1.093	8.811	5	0.925	17.899	5	2.231	16.228	5	1.736	26.510	5	2.783	10.257	5	2.069
6m	13.302	5	2.062	9.911	5	1.739	20.092	5	3.717	18.696	5	3.274	19.335	5	2.802	10.399	5	1.736
9m	9.453	5	0.681	6.965	5	0.771	15.935	5	2.321	14.901	5	1.568	14.983	5	2.316	9.204	5	2.033
12m	4.969	5	0.37	4.170	5	0.288	9.655	5	1.916	9.238	5	2.119	10.880	5	1.029	5.923	5	0.784

group	BLAm ips			BLAm con		
	mean	n	SEM	mean	n	SEM
2w	17.311	5	1.132	11.152	5	1.543
1m	16.893	4	1.336	11.099	4	0.729
3m	20.142	5	2.915	9.220	5	1.89
6m	18.744	5	3.254	12.288	5	2.191
9m	15.441	4	2.499	10.180	4	1.21
12m	8.299	5	0.857	5.799	5	0.458

Table S1. This table, like Table 1 in the main part of the manuscript, shows the optical densities in %. This table is extended by the numbers of individuals (n) analysed for each time point and structure, as well as the standard error of mean (SEM) of each measurement. For more clarity, the table has been divided into 4 parts. The addition "ips" to the column name stands for ipsilateral (region to the injection side) and the addition “con” for contralateral (to the injection side). CPu: Caudate-Putamen complex, GP: Globus pallidus, EP: Entopeduncular nucleus, SN: Substantia nigra, MThal: Medial thalamic nuclei, VThal: Ventral thalamic nuclei, MC: Motor cortex, Acb: Accumbens nucleus, Pn: Pons, VTA: Ventral tegmental area, Hb: Habenular nuclei, Pir: Piriform cortex, BLAm: Basolateral amygdala nuclei.
